# Nox2 Inhibition Regulates Stress Response and Mitigates Skeletal Muscle Fiber Atrophy during Simulated Microgravity

**DOI:** 10.3390/ijms22063252

**Published:** 2021-03-23

**Authors:** John M. Lawler, Jeffrey M. Hord, Pat Ryan, Dylan Holly, Mariana Janini Gomes, Dinah Rodriguez, Vinicius Guzzoni, Erika Garcia-Villatoro, Chase Green, Yang Lee, Sarah Little, Marcela Garcia, Lorrie Hill, Mary-Catherine Brooks, Matthew S. Lawler, Nicolette Keys, Amin Mohajeri, Khaled Y. Kamal

**Affiliations:** 1 Redox Biology & Cell Signaling Laboratory, Department of Health and Kinesiology, Graduate Faculty of Nutrition, Texas A&M University, College Station, TX 77843, USA; jeffrey-hord@uiowa.edu (J.M.H.); patryan412@tamu.edu (P.R.); dsh0008@tamu.edu (D.H.); mariana@alunos.fmb.unesp.br (M.J.G.); dinahrdz@tamu.edu (D.R.); vinicius.guzzoni@gmail.com (V.G.); erika.garcia@tamu.edu (E.G.-V.); c.d.green@tamu.edu (C.G.); leeyang20@gmail.com (Y.L.); selittle@tamu.edu (S.L.); mmg2927@tamu.edu (M.G.); lorrie621@tamu.edu (L.H.); mcbrooks797@tamu.edu (M.-C.B.); lawle172@umn.edu (M.S.L.); nikkikeys@tamu.edu (N.K.); am1@tamu.edu (A.M.); kymoustafa@tamu.edu (K.Y.K.); 2 Department of Molecular Physiology & Biophysics, University of Iowa, Iowa City, IA 52242, USA; 3 Brigham & Women’s Hospital, Harvard Medical School, Cambridge, MA 02115, USA; 4 Department of Kinesiology, University of Texas Rio Grande Valley, Harlingen, TX 78539, USA; 5 Department of Cellular and Molecular Biology, Federal University of Paraíba, João Pessoa, Paraíba 58059900, Brazil; 6 School of Medicine, University of North Carolina, Chapel Hill, NC 27516, USA; 7 Department of Biomedical Engineering, University of Minnesota, Minneapolis, MN 55455, USA

**Keywords:** NADPH oxidase, oxidative stress, unloading, atrophy, skeletal muscle, HSP70, MnSOD, Nrf2, nNOS

## Abstract

Insufficient stress response and elevated oxidative stress can contribute to skeletal muscle atrophy during mechanical unloading (e.g., spaceflight and bedrest). Perturbations in heat shock proteins (e.g., HSP70), antioxidant enzymes, and sarcolemmal neuronal nitric oxidase synthase (nNOS) have been linked to unloading-induced atrophy. We recently discovered that the sarcolemmal NADPH oxidase-2 complex (Nox2) is elevated during unloading, downstream of angiotensin II receptor 1, and concomitant with atrophy. Here, we hypothesized that peptidyl inhibition of Nox2 would attenuate disruption of HSP70, MnSOD, and sarcolemmal nNOS during unloading, and thus muscle fiber atrophy. F344 rats were divided into control (CON), hindlimb unloaded (HU), and hindlimb unloaded +7.5 mg/kg/day gp91ds-tat (HUG) groups. Unloading-induced elevation of the Nox2 subunit p67phox-positive staining was mitigated by gp91ds-tat. HSP70 protein abundance was significantly lower in HU muscles, but not HUG. MnSOD decreased with unloading; however, MnSOD was not rescued by gp91ds-tat. In contrast, Nox2 inhibition protected against unloading suppression of the antioxidant transcription factor Nrf2. nNOS bioactivity was reduced by HU, an effect abrogated by Nox2 inhibition. Unloading-induced soleus fiber atrophy was significantly attenuated by gp91ds-tat. These data establish a causal role for Nox2 in unloading-induced muscle atrophy, linked to preservation of HSP70, Nrf2, and sarcolemmal nNOS.

## 1. Introduction

Skeletal muscles are dynamic metabolic and force-producing tissues that adapt to changes in mechanical loading by remodeling their mass and force-generating capacity. Mechanical unloading that occurs with spaceflight, casting, splint, and bedrest elicits a rapid and profound atrophy of affected skeletal muscles [1], coupled with a partial shift of Type I to Type II muscle fibers [2]. Regulating skeletal muscle size in response to changing, dynamic mechanical stressors is accomplished by altering muscle fiber cross-sectional area (CSA). Although the mechanisms underlying skeletal muscle atrophy are complex, redundant, and multifactorial, emerging data over the last 15 years has focused on upstream oxidative stress, inflammatory signaling, and insufficient stress proteins [3,4,5,6]. Our laboratory identified reduction in heat shock protein 70 (HSP70) and heat shock protein 25 (HSP25) in rat soleus muscles during mechanical unloading [7,8]. Andy Judge and colleagues demonstrated that transfection of *HSP70* and *HSP25* genes [6,9] mitigated unloading-induced muscle atrophy. We recently reported that the combination of curcumin and fish oil produced an elevation of HSP70 and protected against muscle fiber atrophy during unloading [10]. We previously noted suppression of manganese-isoform of superoxide dismutase (MnSOD) during mechanical unloading [8,11].

The contribution of oxidative stress to mechanical unloading-induced muscle atrophy remains complex and not fully understood. While some antioxidant cocktails [12] and nutraceutical antioxidants [13] have been equivocal or ineffective in protecting against atrophy of unloaded skeletal muscle, the overexpression of catalase [14], mitochondrial-targeted antioxidants [15], and intervention with a mimetic of superoxide dismutase and catalase (EUK-134) [4,16] were more successful. We previously noted an elevation of the pro-oxidant enzyme NAPDH oxidase-2 (Nox-2) during unloading in skeletal muscle [16,17]. However, the potential role of Nox2 in skeletal muscle hypertrophy remains poorly understood.

Recently, we discovered that the angiotensin receptor-blocker losartan reduced Nox2 activity and assembly of Nox2 subunits (e.g., p67phox, rac1 and p47phox) in skeletal muscle of rats experiencing 7 days of hindlimb unloading. Inhibition of oxidative stress by EUK-134 or via the angiotensin II receptor 1 inhibition also prevented skeletal muscle fiber atrophy and translocation of the mu-splice variant of nitric oxide synthase (nNOSµ) from the sarcolemma into the cytosol [4,16,17]. In addition, inhibition using the chimeric peptide (gp91ds-tat) has been reported to inhibit p47phox association with gp91phox in NADPH oxidase [18]. Recently, our laboratory discovered that using gp91ds-tat inhibition of Nox2 caused more than a 50% reduction in superoxide produced during unloading [17]. Previously, Suzuki et al. [19] and Vitadello et al. [20] reported a mechanistic connection between nNOSµ translocation and muscle atrophy.

Therefore, the purpose of this investigation was to test the effects of Nox2 inhibition on markers of stress-protective proteins, nNOSµ translocation, and muscle atrophy during mechanical unloading. A peptidyl inhibition of Nox2 was used to prevent the assembly of the Nox2 complex and thus preventing nNOSµ translocation. We hypothesized that Nox2 inhibition would rescue or elevate protein expression HSP70 and manganese-specific superoxide dismutase (MnSOD), mitigate loss of sarcolemmal nNOSµ, and attenuate unloading-induced muscle atrophy.

## 2. Results

### 2.1. Effect of Nox2 Inhibition on the Nox2 Complex Assembly

**Figure 1:**
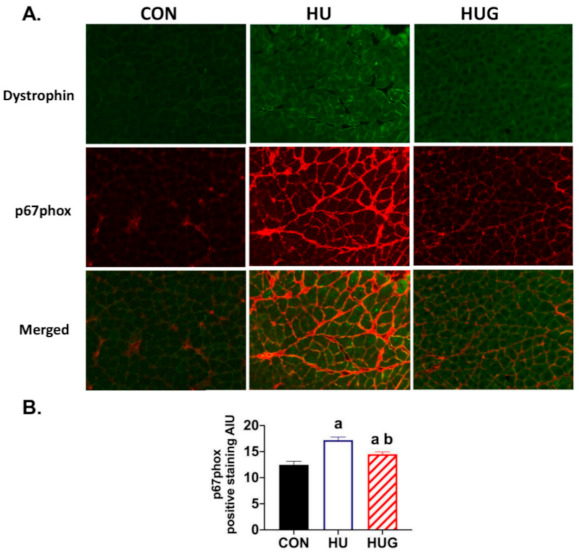
Peptidyl inhibition of NADPH oxidase-2 (Nox2) by gp91ds-tat reduces membrane-positive staining of Nox2 subunit p67phox. (**A**) p67 blot immunofluorescence during hindlimb unloading in soleus muscle, with dystrophin as a membrane marker. (**B**) Positive staining for p67phox was quantified with one-way ANOVAs run to determine mean differences in positive staining. Fischer-344 rats were divided into the following groups (*n* = 6/group): ambulatory controls (CON), hindlimb unloaded + scrambled peptide (HU), and HU + gp91ds-tat (HUG). (a) is statistically significantly different than CON; (b) is significantly different than HU.

To confirm that gp91ds-tat was affecting assembly of the Nox2 complex, we tested the effect of Nox2 inhibition on p67phox-positive staining, including the membrane. P67phox is a cytosolic protein that binds to the Nox2 complex, permitting activation as a source of ROS production [16,17]. We found substantial enhancement of membrane-bound-positive staining with hindlimb unloading, using dystrophin as a sarcolemmal marker (Figure 1). Gp91ds-tat treatment mitigated the enhancement of sarcolemmal-positive staining in the unloaded rat soleus muscle. Quantification of p67phox-positive staining revealed a significant increase in p67phox levels in hindlimb unloaded rats injected with a scrambled sequence. Gp91ds-tat injections, however, significantly attenuated unloading-induced increases in p67phox-positive staining. In addition, the HUG group-positive staining for p67phox did remain significantly higher than ambulatory controls.

### 2.2. Nox2 Inhibition Attenuates Disruption of HSP70 Protein Expression

**Figure 2:**
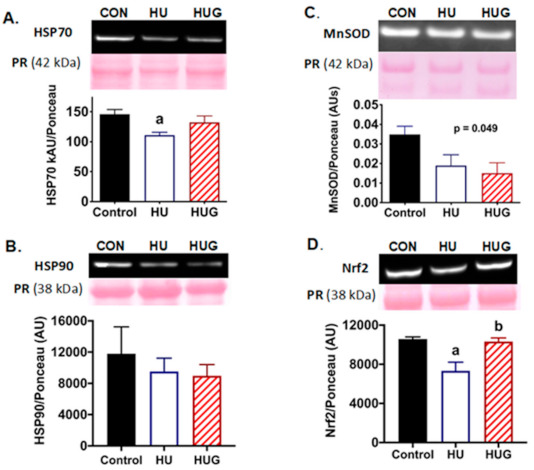
Protein abundance for heat shock protein 70 (HSP70), heat shock protein 90, manganese superoxide dismutase (MnSOD), and nuclear factor erythroid 2-related factor 2 (Nrf2) in ambulatory controls (CON), hindlimb unloaded + scrambled peptide (HU), and HU + gp91ds-tat (HUG). Representative immunoblots with Ponceau stain as a protein loading control are found in (**A**) HSP70, (**B**) HSP90, (**C**) MnSOD, and (**D**) Nrf2. Protein abundance quantification was determined using Western immunoblotting and quantified using ImageJ for HSP70, HSP90, MnSOD, and Nrf2. (a) designates significantly different means than CON; (b) represents a significance difference vs. HU. MnSOD ANOVA was significantly different (*p* = 0.049) with a downward trend for HU and HUG groups compared with Control solei.

HSP70 protein abundance was assessed. Not surprisingly, there was a significant reduction in HSP70 levels with unloading (Figure 2A). However, protein expression levels in the unloading group receiving gp91dstat were not significantly different than the control group. We also quantified protein abundance of HSP90 (Figure 2B). While HSP90 trended lower with hindlimb unloading in the rat soleus, there were no statistically significant effects for either unloading or Nox2 inhibition.

### 2.3. Nrf2 Levels Are Elevated by the Inhibition of Nox2

MnSOD is a mitochondrial antioxidant enzyme that we previously discovered is suppressed with unloading [8]. MnSOD levels here trended significantly lower with unloading (Figure 2C) with no significant protection by Nox2 inhibition. Nuclear factor erythroid 2-related factor 2 (Nrf2) is a leucine zipper protein and transcription factor that regulates antioxidant proteins including superoxide dismutase, catalase, heme oxygenase-1, and glutathione peroxidase [21]. We observed that Nrf2 abundance in the nuclear fraction was significantly reduced by unloading (Figure 2D). In contrast, Nrf2 levels were significantly protected during unloading when Nox2 was inhibited, suggestive of potential for preservation of antioxidant protection.

### 2.4. Nox2 Inhibition Mitigates the Reduction in nNOS Activity and Translocation

**Figure 3:**
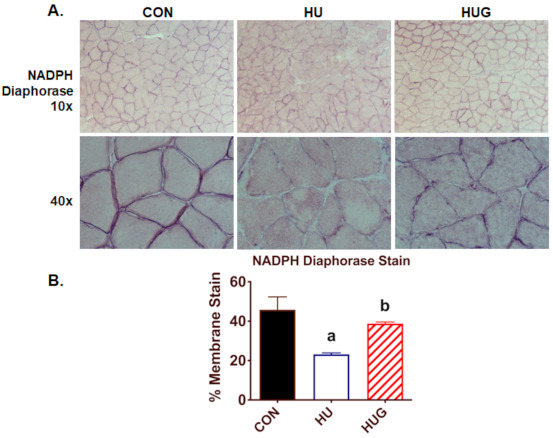
NADPH diaphorase staining assay, a biomarker of nNOS activity, for the rat soleus muscle is displayed in (**A**). (**B**) NADPH diaphorase-positive staining was quantified for ambulatory controls (CON), hindlimb unloaded with scrambled peptide (HU), and HU + gp91ds-tat (HUG). To distinguish among statistically different group means, (a) designates a significant difference vs. CON; (b) indicates significance difference with the HU group.

**Figure 4:**
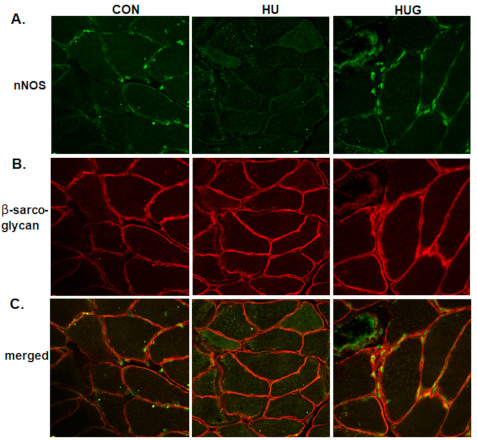
Confocal fluorescence staining for neuronal nitric oxide synthase is displayed in Figure 4. nNOS images are found in (**A**). β-sarcoglycan was used as a membrane marker in (**B**). Merged images are shown in (**C**). Fluorescence stains for the following groups were taken for ambulatory controls (CON), hindlimb unloaded + scrambled peptide (HU), and HU with gp91ds-tat peptidyl inhibitor of Nox2 (HUG). nNOS-positive staining subsides with HU.

In order to identify changes in nNOS activity and thus •NO bioavailability, we used the NADPH diaphorase assay technique, previously described by Hord et al. [20]. Here, we found a profound decrease in NADPH diaphorase-positive staining (Figure 3), with loss of nNOS activity most apparent visualized around the cell membrane. In contrast, gp91ds-tat provided substantial and significant protection against the unloading-induced reduction in NADPH diaphorase-positive staining. We then visualized changes in sarcolemmal nNOS using confocal fluorescence microscopy, using β-sarcoglycan as our stable sarcolemmal marker. nNOS-positive staining at the sarcolemmal was largely and heterogeneously lost in the unloaded soleus (Figure 4) but was mitigated by gp91ds-tat, consistent with NADPH diaphorase findings.

### 2.5. Changes in Skeletal Muscle Morphology

**Figure 5:**
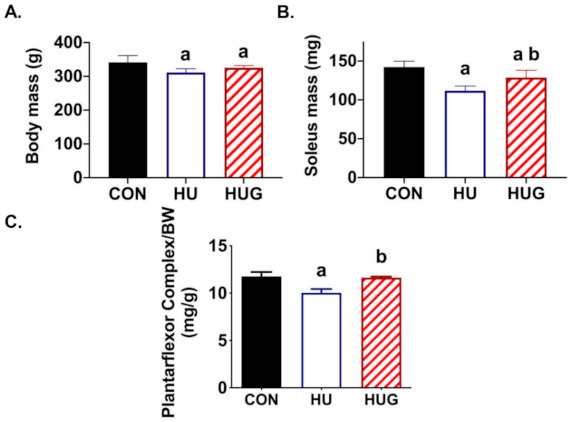
Effects of peptide Nox2 inhibition on skeletal muscle and body mass. (**A**) Body mass for ambulatory controls (CON), hindlimb unloaded + scrambled peptide (HU), and HU + gp91ds-tat (HUG). (**B**) Soleus mass means are. (**C**) Plantar flexor mass/body mass ratio. (a) designates significantly different means than CON; (b) represents a significance difference vs. HU.

To determine whether Nox2 inhibition protected skeletal muscle morphology, we measured soleus mass, plantar flexor weight, muscle fiber cross-sectional area, and muscle fiber type. Body weight decreased modestly, but significantly in both HU and HUG groups (approximately 15–20 g). Soleus mass was 24% lower in unloaded F344 rats, while significantly protected in gp91ds-tat-treated rats (Figure 5). However, soleus mass in the HUG group remained significantly lower in than control, ambulatory rats. Plantar flexor complex mass, corrected for body mass, was significantly reduced in unloaded rats injected with the scramble peptide sequence. However, it was significantly protected by gp91ds-tat and not significantly lower than in the control group.

**Figure 6:**
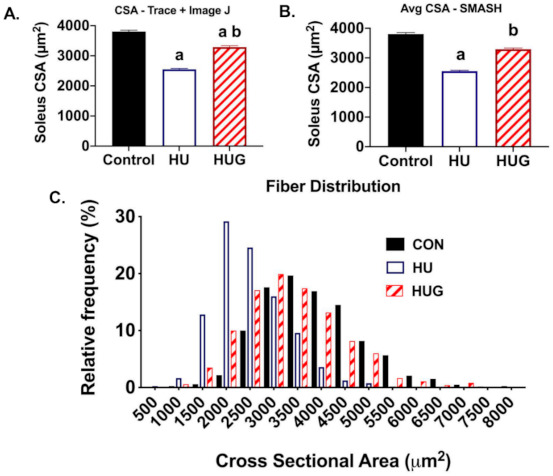
Nox2 inhibition via gp91ds-tat reduces unloading-induced soleus fiber atrophy. Ambulatory controls (CON), hindlimb unloaded + scrambled peptide (HU), and HU + gp91ds-tat (HUG) groups are displayed. (**A**) Soleus fiber cross-sectional area (CSA) was quantified using a hand traced macro on ImageJ. (**B**) We also quantified muscle fiber using the SMASH program. (**C**) Muscle fiber CSA distribution. To distinguish among groups with statistically significant group means, (a) is significantly different than CON; (b) is significantly different than HU.

Soleus muscle fiber cross-sectional area (CSA) was assessed using two methods: (1) hand-tracing images and subsequent ImageJ analysis (Figure 6A); and (2) programed quantification using the SMASH program (Figure 6B), adapted by our laboratory as described earlier [10]. We also calculated muscle fiber CSA, divided into 500 µm^2^ divisions or modules (Figure 6C). We found a significant decrease in soleus CSA due to unloading in rats injected with the scrambled peptide sequence using both the hand trace methodology (−35%) and SMASH (−37%). However, there was a significant attenuation of muscle fiber atrophy by gp91dstat using both methodologies. Muscle fiber CSA did remain significantly lower in the HUG group than controls using hand trace methodology. However, there was a trend without statistical significance downward in HUG muscle fiber CSA vs. controls using SMASH.

We then subdivided muscle fiber CSA into 500 µm^2^ increments to develop histogram blots for CON, HU, and HUG groups. Upon analysis we observed a significant left shift in soleus muscle fiber CSA in Figure 6C, signifying a substantially greater number of small muscle fibers (<2500 µm^2^). In contrast, gp91ds-tat treatment prevented or mitigated the large left shift in muscle fiber CSA.

**Figure 7:**
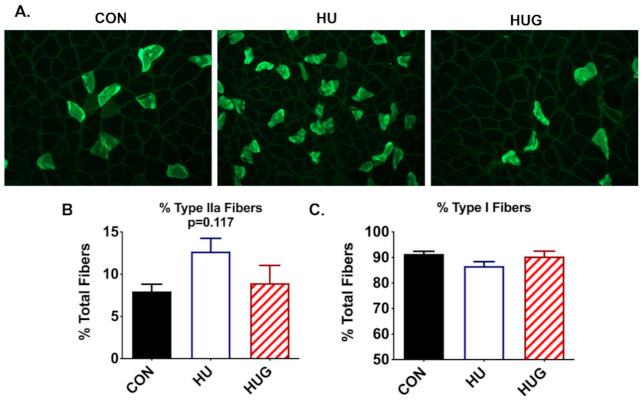
Immunofluorescence stains for Type IIa fibers using Hybridoma antibodies is shown in (**A**) % Type IIa and Type I fibers were quantified for ambulatory controls (CON), hindlimb unloaded + scrambled peptide (HUSCr), and HU + gp91ds-tat (HUG) in (**B**,**C**). Hindlimb unloading results in a trend upwards in Type IIa fiber but did not reach statistical significance.

The percentage of Type II fibers trended higher in HU rats but did not research statistical significantly. (Figure 7) HUG levels of % Type II and % Type I fibers were similar to controls. The trend downward in the % of Type I fibers during hindlimb unloading did not reach statistical significance.

## 3. Discussion

**Figure 8:**
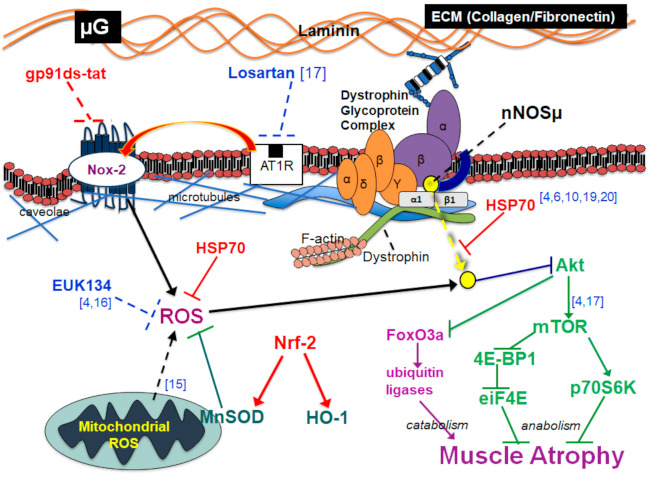
Summary model of Nox2 inhibition (in red) and downstream effects. The figure includes integration of findings from previous studies discussed within the manuscript [4,6,10,15,16,17,19,20].

Inhibition of Nox2 with the gp91ds-tat peptide resulted in partial protection of HSP70 and Nrf2 protein abundance in the rat soleus during mechanical unloading compared with hindlimb unloading with the scrambled sequence. However, Nox2 inhibition did not mitigate the loss on the antioxidant enzyme MnSOD. nNOS bioavailability at the sarcolemma was significantly protected by Nox2 inhibition. As predicted, abatement of nNOS translocation by gp91ds-tat was linked to partial protection of soleus mass and muscle fiber cross-sectional area during unloading. These data indicate that Nox2 is causal in unloading-induced nNOS translocation and muscle fiber atrophy, potentially mediated in part by a decline in HSP70. A summary slide of our model outlining the principle findings and following discussion is presented in Figure 8.

The importance of a loss or insufficiency in HSP70, and other stress-protective proteins, serving as a mediator of unloading-induced muscle atrophy has been supported by multiple independent investigations [6,7,8,20,22]. While the mechanisms underlying protection against atrophy are not completely understood, the role of HSP70 as a protein chaperone and antioxidant has gained significant support [6,7]. Indeed, transfection of the *HSP70* gene results in a significant attenuation of unloading or casting-related muscle atrophy [6]. The stress-protective protein grp94 has been causally linked to partial abrogation of nNOS translocation and muscle atrophy [20,23], including upregulation of grp94 via curcumin supplementation [24]. Protection against skeletal muscle atrophy during unloading by combining fish oil and curcumin supplementation was recently linked to a concomitant elevation of HSP70 [10]. Reduction in HSP70 with aging may also contribute to sarcopenia [25]. However, to our knowledge this is the first data to suggest that an unloading-induced suppression of HSP70 could contribute to loss of sarcolemmal nNOS. This important notion requires future examination.

Nox2 is a membrane oxidoreductase that produces ROS in response to skeletal muscle contractions [26] and stretch [27]. Extracellular ROS appear to communicate with neighboring myocytes, thus regulating contractile function [28,29], glucose uptake [30,31], pre-conditioning elevation of stress-protective proteins [32], metabolism [33], and gene expression [34]. However, the assembly of the Nox2 complex dramatically rises with Duchenne muscular dystrophy [35] and Type II diabetes [36], contributing to pathology. Paradoxically, there has been an observed elevation of Nox2 with mechanical unloading and denervation in skeletal muscle [16,37].

Thus, we tested the ability of a peptidyl inhibitor of Nox2 to affect skeletal muscle morphology. Indeed, Nox2 inhibition significantly mitigated skeletal muscle fiber atrophy, establishing a causal role for Nox2 in skeletal muscle atrophy. Recently, our laboratory tested the ability of angiotensin II receptor I (AT1R) blockade (ARB) to mitigate elevation of Nox2 assembly, and thus oxidative stress, nNOS translocation and muscle fiber atrophy [17]. Indeed, the ARB losartan attenuated unloading-induced elevation of Nox2 subunits (gp91phox, p67phox, p47phox, Rac1), Nox2 activity, loss of sarcolemmal nNOS, and muscle fiber atrophy [17]. Together, these data indicate that an AT1R–Nox2 pathway is contributing to unloading-induced atrophy in skeletal muscle.

The causal nature of Nox2 on nNOS translocation and muscle fiber atrophy supports previous evidence of the role of oxidative stress in mechanotransduction and atrophic process during mechanical unloading. Overexpression of the antioxidant enzyme catalase, with localization in mitochondria, previously suppressed ubiquitin ligase activation and skeletal muscle atrophy [14]. Our laboratory previously used a salen-manganese mimetic of superoxide dismutase and catalase [4,16] to demonstrate that oxidative stress triggers translocation of nNOS, activation of FoxO3a, and suppression of anabolic signaling thus leading to muscle atrophy [4,16]. Together, these data suggest that AT1R- and Nox2-mediated oxidative stress is important in mechanotransduction during mechanical unloading in skeletal muscle.

Our data also indicate a potential interaction between Nox2 and HSP70 during unloading. Previous studies in other tissues or pathologies revealed that HSP70 can regulate NADPH oxidases, including Nox2, and thus oxidative stress [38,39]. HSP70, through the carboxy terminus of Hsp70 interacting protein (CHIP), caused ubiquination and thus degradation of NADPH oxidase-4 (Nox4) in kidney cells [39]. Interestingly, losartan enhanced HSP70 while genetic ablation of HSP70 elevated NADPH oxidase activity. HSP70 may also suppress ROS production by inhibiting the pro-inflammatory transcription factor NF-kappaB [38]. However, the interaction between HSP70 and Nox2 in skeletal muscle is complex and not well understood in wasting skeletal muscle [37].

The antioxidant enzyme MnSOD was downregulated with mechanical unloading, consistent with previous studies [8,11]. However, inhibition of Nox2 did not prevent loss of MnSOD during unloading. Given that mitochondrial ROS are also elevated during mechanical unloading in skeletal muscle [15], our data suggest that MnSOD inhibition may be more closely linked to mitochondrial ROS rather than Nox2 during unloading.

The antioxidant transcription factor Nrf2 has been shown to be downregulated with mechanical unloading [40], while providing protection against muscle wasting and oxidative stress with hibernation [41], sarcopenia [42,43], and TNF-alpha-induced atrophy [44]. However, it is uncertain whether protection of Nrf2 when Nox2 was inhibited in the current study contributed to the protection against unloading-induced skeletal muscle fiber atrophy [40]. Interestingly, during hyperthermia Nrf2 may be involved in upregulating HSP70 and HO-1 [45]. Nrf2 is a critical regulator of HO-1 through binding the antioxidant response element (ARE), which activated by the extracellular regulated kinase (ERK) [46]. Conversely, HO-1 can also upregulate Nrf2 through the inhibition or ablation of p38 MAPK [47,48]. However, the mechanisms underlying Nrf2 regulation of HSP70 during unloading and inhibition of Nox2 remain unknown.

Quercetin was recently found to protect cytokine-linked atrophy by elevating Nrf2 and downstream HO-1 [44]. Other nutritional supplements such as fish oil and curcumin appeared to provide protection against unloading-induced muscle atrophy through upregulation of HSP70 and downregulation of Nox2 [10]. Our data suggest that protection of HO-1 could be more important than MnSOD in protecting against unloading-induced atrophy and, while unresolved, is a focus of future investigations. Future experiments could use MAPK inhibitors, which upregulate Nrf2/HO-1 pathway, as novel pharmacological therapeutics against unloading-induced atrophy.

In summary, inhibition of Nox2 during mechanical unloading protected HSP70 and Nrf2 levels, attenuated translocation of nNOS from the sarcolemma, and reduced muscle fiber atrophy. The data provide the first evidence that elevated Nox2 can play a causal role in mechanotransduction and remodeling that occur in unloaded skeletal muscle. Future research should further examine interaction of redox signaling and stress-protective proteins in unmodeled skeletal muscle.

## 4. Material and Methods

### 4.1. Animals

Four-month-old Fisher-344 (F344) rats were used in this investigation and were purchased from Harlan Sprague-Dawley. F344 rats are a common rodent model for mechanical unloading and spaceflight. Phenotypic muscle fiber trophy and fiber type shift from slow to fast twitch are similar to humans [49]. Rats were kept in a 12/12 h daylight cycle, temperature controlled, and provided chow and water ad libitum. Animal care met all federal stipulations per NIH statutes (NRC Guide for care and use of lab animals, 8th Edition, 2011) and consistent with the Animal Welfare Act and the Public Health Service Policy as well as the Humane Care and Use of Laboratory Animals.

### 4.2. Experimental Design and Hindlimb Unloading

Fisher-344 rats were divided into three groups (*n* = 6/group): ambulatory controls (CON), hindlimb unloaded (7 days) injected i.p. with a scrambled sequence as a control (HUScr), and hindlimb unloaded +7.5 mg/kg gp91ds-tat (HUG). Rats were injected beginning 24 h before the experiment. Scrambled sequence (sgp91ds-tat) TAMRAGGGGYGRKKRRQRRRCL-RITRQSRNH2 and active Nox2 inhibitor gp91ds-tat ([H]RKKRRQRRRCSTRIRRQL-[NH_3_]) were purchased from Bio-synthesis (Lewisville, TX, USA).

The hindlimb unloading procedure was conducted as previously described [4]. Hindlimb unloading replicates the physiological effects of bedrest and spaceflight [17]. Briefly, rats were anesthetized using a cocktail of ketamine (75 mg/kg) and xylazine (10 mg/kg), and tails were cleaned with alcohol. Rats were then fitted with a flexible harness attached to a crosswire through a swivel that allowed ambulation around the cage and free access to food and water. Rats were then elevated at a spinal angle of 40° so that hindfeet were approximately 1 cm off the cage floor. At the end of the hindlimb unloading period, rats from all groups were sacrificed with sodium pentobarbital (120 mg/kg), soleus muscles were dissected, cleaned, and weighed. Muscle samples were frozen in isopentane cooled in liquid nitrogen.

### 4.3. Muscle Tissue Preparation

Soleus samples were minced and homogenized in lysis buffer (pH = 7.4) containing 25 mM Tris-HCL, pH 7.4, 100 mM NaCl, 1 mM EDTA, 1 mM EGTA, as well as a protease and phosphatase inhibitor cocktail. The homogenization procedure involved a procedure previously described [17,50]. Briefly, solei muscles were homogenized at 4 °C in lysis buffer using a motorized ground glass on ground glass mortar and pestle system. Debris was pelleted by centrifugation at 1000× *g* for 10 min. The resulting supernatant was centrifuged at 20,000× *g*.

### 4.4. Western Immunoblot

Protein expression was determined by Western immunoblot analysis per the technique outlined previously [4]. The 20 µg samples of soleus were loaded on 10% polyacrylamide gels and electrophoresed using a Bio-Rad Protein III gel-box onto a nitrocellulose membrane (Bio-Rad, Hercules, CA, USA). Soleus extracts (20 µg) along with sample buffer were loaded into wells of 10% SDS-PAGE gels. Samples were then electrophoresed at 120 V for 75 min. Membranes were blocked in a non-fat milk buffer (5% non-fat milk in TBS) for 1 h. Following blocking, membranes were incubated overnight (4 °C) in blocking buffer with the appropriate primary antibodies: anti-MnSOD (rabbit pAb; 1:20,000; Cat# ab13533, Abcam, Cambridge, MA, USA), anti-Nrf2 (rabbit pAb; 1:1000; Cat# 16396-1-AP, Proteintech, Rosemont, IL, USA), anti-HSP70 (rabbit pAb; 1:2000; Cat# ADI-SPA-812-F, Enzo, Farmingdale, NY, USA), and anti-HSP90 (rabbit mAb; 1:1000; Cat# C45G5, Cell Signaling, Danvers, MA, USA).

Proteins were visualized by Super Signal West Dura Extended Duration Substrate (Cat# 34076, Thermo Scientific, Waltham, MA, USA) enhanced chemiluminescence detection, and developed with a Fuji LAS-4000 Luminescent Image Analyzer (FujiFilm Medical Systems, Lexington, MA, USA). Quantification was performed using NIH ImageJ software with Ponceau-S staining (cytoplasmic: band ~38 kDa mark (GAPDH)) used as a loading control.

### 4.5. Histological Analysis

Soleus muscle samples were cut in 10 µm sections from the midbelly of the muscle using a cryostat (Thermo Scientific, Shandon Cryotome FSE) and allowed to air dry for 30 min. We used NADPH diaphorase histochemical analysis described by Hord, et. al [17] to ascertain the presence and localization of active nNOS. Soleus muscle cross-sections were fixed for 20 min with 2% paraformaldehyde, then washed 2X in PBS. Slides were then incubated at 37 °C for two hours in 50 mM Tris-HCl buffer (pH = 8.00) with 0.2% Triton-X-100, 0.5 mM nitrotetrazolium blue chloride, and 1 mM β-NADPH. Distilled water was used to stop the enzymatic reaction, and slides were then air dried. Soleus sections were then mounted with Vectamount permanent medium (Vectamount, Cat# H-5000, Vector Laboratories, Burlingame, CA, USA).

Images of stained were taken using a Zeiss Axioplot upright microscope and Zeiss Axiocam HRc color camera (Carl Zeiss Microimaging, Thornwood, NY, USA). NIH Image J was used to quantify NADPH-diaphorase-stained samples ImageJ by using the cross-sectional circumference (CSC) to quantify the percentage of the positively stained sarcolemma.

### 4.6. Immunofluorescence (IF)

Soleus samples were sectioned at a thickness of 10 µm in a cryostat (Thermo-Fisher: Shandon Cryotome FSE) at −15 °C onto a microscope slide [4]. Sections were fixed in acetone for 10 min. Sections were blocked in TBS with 0.05% Tween20 and 10% goat serum for 15 min. Primary antibodies were incubated for 60 min in blocking buffer for p67phox (mouse mAb; 1:200; BD Biosciences 610913) and dystrophin (rabbit pAb; 1:200; Cat# sc15376, Santa Cruz Biotechnology Inc., Dallas, TX, USA). Appropriate 2° antibodies (1:200 dilution) with a fluorochrome (e.g., AlexaFluor 488) were attached. Hybridoma antibodies for MHCI, MHCIIA, and MHCIIB were used for fiber type.

Slides were then mounted with ProLong Gold antifade reagent (ProlongTM, Cat# P36930, Life Technologies, Eugene, OR, USA) and visualized on a Zeiss (510 META) confocal laser scanning microscope (CLSM), or via fluorescence microscope (Zeiss Axioplan, Carl Zeiss, Gottingen, Germany) in the Texas A&M Image Analysis Core Facility. Images were analyzed using membrane macros for Fiji ImageJ processing [51] and the SMASH program for specific membrane protein abundance. Sarcolemmal proteins were co-localized with β-sarcoglycan or Na+/K+ ATPase to visualize the sarcolemma as membrane marker. A subset of samples was used for Type I CSA (~160 fibers/muscle) and Type II CSA analysis (~50 fibers/muscle). All images were calibrated with a stage micrometer and cross-sectional area is presented as µm^2^. We assessed > 200 muscle fibers per muscle sample in all groups for muscle fiber cross-sectional area.

### 4.7. Statistical Analysis

Summary data are reported as the mean ± SEM. One-way ANOVAs with Student–Newman Keuls use post hoc on GraphPad 8 software (GraphPad Software, Inc., San Diego, CA, USA) were used to distinguish among group mean differences. Skeletal muscle fiber cross-sectional area was evaluated with a nested ANOVA design with Tukey’s post hoc test utilized to distinguish among group differences. Level of statistical significance was set at *p* < 0.05.
